# Pharmacogenetic analysis of high-dose methotrexate treatment in children with osteosarcoma

**DOI:** 10.18632/oncotarget.11543

**Published:** 2016-08-23

**Authors:** Marta Hegyi, Adam Arany, Agnes F. Semsei, Katalin Csordas, Oliver Eipel, Andras Gezsi, Nora Kutszegi, Monika Csoka, Judit Muller, Daniel J. Erdelyi, Peter Antal, Csaba Szalai, Gabor T. Kovacs

**Affiliations:** ^1^ Second Department of Pediatrics, Semmelweis University, H-1094 Budapest, Tűzoltó utca 7-9, Hungary; ^2^ Department of Measurement and Information Systems, University of Technology and Economics, H-1111 Budapest, Műegyetem rkp. 3, Hungary; ^3^ Department of Organic Chemistry, Semmelweis University, H-1092 Budapest, Hőgyes Endre u. 7, Hungary; ^4^ Department of Genetics, Cell and Immunobiology, Semmelweis University, H-1089 Budapest, Nagyvárad tér 4, Hungary

**Keywords:** osteosarcoma, methotrexate, toxicity, SNP, Bayesian network-based Bayesian multilevel analysis of relevance (BN-BMLA)

## Abstract

Inter-individual differences in toxic symptoms and pharmacokinetics of high-dose methotrexate (MTX) treatment may be caused by genetic variants in the MTX pathway. Correlations between polymorphisms and pharmacokinetic parameters and the occurrence of hepato- and myelotoxicity were studied. Single nucleotide polymorphisms (SNPs) of the *ABCB1*, *ABCC1*, *ABCC2*, *ABCC3*, *ABCC10*, *ABCG2*, *GGH*, *SLC19A1* and *NR1I2* genes were analyzed in 59 patients with osteosarcoma. Univariate association analysis and Bayesian network-based Bayesian univariate and multilevel analysis of relevance (BN-BMLA) were applied. Rare alleles of 10 SNPs of *ABCB1*, *ABCC2*, *ABCC3*, *ABCG2* and *NR1I2* genes showed a correlation with the pharmacokinetic values and univariate association analysis. The risk of toxicity was associated with five SNPs in the *ABCC2* and *NR1I2* genes. Pharmacokinetic parameters were associated with four SNPs of the *ABCB1, ABCC3, NR1I2*, and *GGH* genes, and toxicity was shown to be associated with *ABCC1* rs246219 and *ABCC2* rs717620 using the univariate and BN-BMLA method. BN-BMLA analysis detected relevant effects on the AUC_0-48_ in the following SNPs: *ABCB1* rs928256, *ABCC3* rs4793665, and *GGH* rs3758149. In both univariate and multivariate analyses the SNPs *ABCB1* rs928256, *ABCC3* rs4793665, *GGH* rs3758149, and *NR1I2* rs3814058 SNPs were relevant. These SNPs should be considered in future dose individualization during treatment.

## INTRODUCTION

Osteosarcoma is one of the most frequent primary malignant bone tumors that occurs in childhood [1]. Intensive chemotherapy was introduced in the 1970s, which significantly improved survival and currently about two thirds of patients recover [2]. The treatment aims to reduce the initial tumor volume and to eliminate the potential of micrometastases [3]. The cytostatic drugs that are used in preoperative chemotherapy are doxorubicin, cisplatin, ifosphamide, and methotrexate (MTX) [4]. MTX, a folic acid antagonist, is used in high dosages to treat all osteosarcoma tumors. It penetrates into the cells and inhibits the production of metabolites essential for the synthesis of nucleotides [5]. MTX is very effective in the treatment of osteosarcoma; however, reduced elimination may induce side effects such as hepatotoxicity and myelotoxicity. Therefore, MTX-serum levels after administration are routinely monitored together with hepatic and bone marrow function.

There are large inter-individual differences in the elimination of MTX and in the severity of the toxic symptoms during therapy. There are many influencing factors already known and proven, e.g., hydration, pH level of urine, leucovirin dosage, anatomic and physiological parameters, and differences in the protocol. Beyond these, the individual differences in the pharmacokinetics and toxicities are also influenced by genetic factors; however, mapping of the significance of genetic factors influencing MTX metabolism is still in the initial stages. The genetic variants of the relevant genes may alter adsorption, distribution, metabolism, as well as excretion of the drug, and can lead to these differences [6]. A review of Ferrari S showed that effective molecular-targeted therapies are unlikely to be available in the near future due to genomic complexity and the heterogeneity of osteosarcoma. Therefore, optimizing the use of the current drugs available by personalizing chemotherapy treatment is a potential new line for clinical research in osteosarcoma [7].

**Figure 1:**
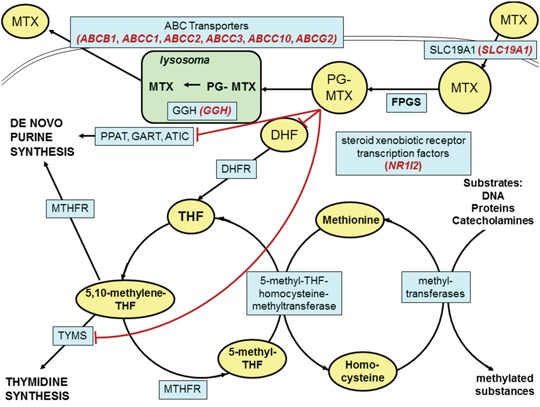
Schematic overview of the intracellular methotrexate pathway Key molecules and derivatives of the pathway are denoted as yellow ovals; transporter proteins and regulatory enzymes as blue rectangles. The examined genes are shown in brackets with red italics near the name of the encoded proteins. MTX: methotrexate; PG-MTX: methotrexate polyglutamates; DHF: dihydrofolate; THF: tetrahydrofolate; FPGS: folylpolyglutamate synthetase; DHFR: dihydrofolate reductase; MTHFR: 5,10-methylenetetrahydrofolate reductase; TYMS: thymidylate synthetase; PPAT: phosphoribosyl pyrophosphate amidotransferase; GART: glycinamide ribonucleotide formyltransferase; ATIC: 5-aminoimidazole-4-carboxamide ribonucleotide formyltransferase; GGH: gamma-glutamyl hydrolase; SLC19A1: solute carrier family 19, member 1; NR1I2: nuclear receptor subfamily 1, group I, member 2; ABC transporters: ATP-binding cassette transporters.

A schematic overview of the intracellular MTX pathway with our selected genes is presented in Figure 1. MTX enters the cell via the solute carrier family 19, member 1 (SLC19A1) transporter protein [5]. A frequently studied polymorphism of this gene is the 80G>A (rs1051266), which leads to an Arg27His exchange in the first transmembrane domain and causes an increased affinity for folic acid [8]. This SNP is associated with the development of severe mucositis in children with osteosarcoma [9]. Within the cell, MTX is polyglutamated by the enzyme folylpolyglutamate-synthetase (FPGS) to its active form, which enters the folic acid cycle. The polyglutamated MTX derivatives are hydrolyzed, creating the monoglutamated form by gamma-glutamyl-hydrolase (GGH) in the lysosome to facilitate elimination [10]. The *GGH* -401C>T (rs3758149) variant enhances promoter activity, increasing protein expression. Increased levels of GGH lead to a decreased accumulation of polyglutamated MTX and MTX-resistance [11]. Free MTX is exported from the cell through the ABCC1-5 and ABCG2 transporters of the ABC (ATP-binding cassette) protein family [12]. The ABCB1 transports its substrates towards the lumen in the intestines and the blood vessels thus preventing adsorption. It participates in excretion, and in forming blood-tissue barriers in the hepatocytes and the cells of the renal tubules [13]. The clinical response to conventional chemotherapy in osteosarcoma is limited due to drug resistance, caused by the overexpression of ABCB1. A study of an ABCB1/ABCC1 inhibitor found that in osteosarcoma cell lines, the inhibitor was able to revert the ABCB1/ABCC1-mediated resistance against doxorubicin [14]. ABCC1 mainly appears in dividing cells and has an explicit barrier function. The primary task of this protein is the protection of cells against toxic effects and contribution to the barrier function [15]. ABCC2 is mainly expressed on the apical surface of hepatocytes, in the intestine, and in the proximal tubules of the kidney [16]. It plays an important role in eliminating endogenous metabolites and xenobiotics. ABCC3 participates in biliary and intestinal excretion of organic anions [17]. ABCG2 blocks the adsorption of toxic substances in the intestine and enhances excretion into the gall bladder [18]. Genetic variants in ABC-transporter genes may alter gene expression, as well as substrate recognition, activity, and function of these proteins [19].

*NR1I2*, alias *SXR* (nuclear receptor subfamily 1, group I, member 2 or steroid and xenobiotic receptor) gene belongs to the nuclear receptor superfamily. The NR1I2 protein activates the transcription of many other genes that participate in the metabolism and secretion of potentially harmful xenobiotics, drugs, and endogenous chemical compounds. In the development of drug resistance, it participates in the regulation of gene transcription [20], [21].

The aim of this study was to investigate the pharmacokinetics of high-dose MTX treatment and the pharmacogenetics, pharmacokinetics, and toxicity of chemotherapy.

## RESULTS

In our analysis, 551 MTX blocks in 59 patients were tested. The genotype and allele frequency of these SNPs are summarized in Table 1.

**Table 1:** Selected genes and single nucleotide polymorphisms important in the methotrexate pathway

Gene	Polymorphism	Allele (1/2)	Genotype N 11 (%) ^1^	Genotype N 12 (%) ^1^	Genotype N 22 (%) ^1^	MAF*100 ^2^ (%)	HWE ^3^ (p)
ABCB1	rs1045642	C/T	16	23	15	49	0.27
ABCB1	rs1128503	C/T	16	29	14	48	0.28
ABCB1	rs9282564	A/G	31	10	0	12	0.37
ABCC1	rs4148358	G/A	32	11	2	17	0.42
ABCC1	rs246219	G/A	38	9	1	11	0.59
ABCC1	rs246221	A/G	25	17	5	29	0.42
ABCC1	rs12922588	A/G	20	23	9	39	0.49
ABCC1	rs215060	A/G	28	18	0	20	0.1
ABCC1	rs4148330	A/G	17	22	4	35	0.4
ABCC2	rs2273697	G/A	32	19	2	22	0.68
ABCC2	rs3740066	G/A	23	21	8	36	0.39
ABCC2	rs717620	G/A	28	13	1	18	0.72
ABCC3	rs4793665	T/C	17	30	9	43	0.48
ABCC3	rs2107441	A/G	16	23	6	39	0.61
ABCC3	rs2412333	G/A	23	24	4	31	0.5
ABCC3	rs733392	G/A	21	28	3	33	0.1
ABCC3	rs12602161	A/G	37	14	0	14	0.1
ABCC10	rs1214748	G/A	15	28	5	40	0.12
ABCC10	rs831314	A/G	37	13	2	16	0.53
ABCC10	rs1214752	G/A	19	18	8	38	0.31
ABCG2	rs2231142	C/A	39	13	0	13	0.3
GGH	rs3758149	C/T	20	28	7	38	0.56
SLC19A1	rs1051266	A/G	15	27	14	49	0.79
SXR	rs7643038	A/G	16	20	9	42	0.55
SXR	rs3814055	G/A	18	23	11	43	0.47
SXR	rs1054190	G/A	36	12	0	13	0.32
SXR	rs3732361	G/A	13	24	9	46	0.72
SXR	rs3814058	A/G	25	16	3	25	0.84
SXR	rs6785049	A/G	11	29	9	48	0.19

SNP: single nucleotide polymorphisms

MTX: methotrexate.

^1^ 11: homozygous for the first allele, 12: heterozygous; 22: homozygous for the second allele.

^2^ MAF: Minor allele frequency * 100 (%).

^3^ HWE: deviation from the Hardy–Weinberg equilibrium (χ2-test).

### Pharmacokinetics of MTX

The results of the univariate association analysis are summarized in Table 2.

**Table 2:** Relevant p-values of univariate association test between SNPs and target variables

Gene	Polymorphism	T_α1/2_	AUC_0-48_	Peak MTX	48 h MTX	Hepato-toxicity	Myeol-toxicity
ABCB1	rs1045642	-	-	-	-	-	-
ABCB1	rs1128503	-	-	-	-	-	-
ABCB1	rs9282564	-	↑0.04	↑0.02	-	-	-
ABCC1	rs4148358	-	-	-	-	-	-
ABCC1	rs246219	-	-	-	-	-	-
ABCC1	rs246221	-	-	-	-	-	-
ABCC1	rs12922588	-	-	-	-	-	-
ABCC1	rs215060	-	-	-	-	-	-
ABCC1	rs4148330	-	-	-	-	-	-
ABCC2	rs2273697	-	-	-	-	-	↑0.02
ABCC2	rs3740066	-	↓0.01	-	-	-	↓0.02
ABCC2	rs717620	-	-	-	-	-	-
ABCC3	rs4793665	-	↓0.03	-	-	-	-
ABCC3	rs2107441	-	-	-	-	-	-
ABCC3	rs2412333	-	-	-	-	-	-
ABCC3	rs733392	-	-	-	-	-	-
ABCC3	rs12602161	-	-	-	-	-	-
ABCC10	rs1214748	-	-	-	-	-	-
ABCC10	rs831314	-	-	-	-	-	-
ABCC10	rs1214752	-	-	-	-	-	-
ABCG2	rs2231142	↑0.037	-	-	-	-	-
GGH	rs3758149	-	-	-	-	-	-
SLC19A1	rs1051266	-	-	-	-	-	-
SXR	rs7643038	↑0.02	-	-	-	-	-
SXR	rs3814055	↑0.04	-	-	-	-	-
SXR	rs1054190	-	-	-	-	-	-
SXR	rs3732361	-	-	-	↑0.01	↓0.014	↓0.013
SXR	rs3814058	-	-	-	-	↓0.007	↓0.007
SXR	rs6785049	-	-	-	↑0.006	↓0.02	↓0.01

“-” no correlation with the number of variant allele and the target variable (*p* > 0.05).

Direction of the arrow: the presence of the variant allele increases (↑) or decreases (↓) the value of the pharmacokinetic target variable or the frequency of the toxicity.

Analyzing the half-live of MTX, it seems that the presence of a variant allele of *ABCG2* rs2231142 (odds ratio (OR) = 4.2, 95% confidence interval (CI) = 1.0–17.8, *p* = 0.037) is associated with a longer first half-life of MTX (T_α1/2_). Moreover, the presence of the SNPs *NR1I2* rs7643038 (OR = 2.6, 95% CI = 1.2–5.9, *p* = 0.02) and rs3814055 (OR = 2.2, 95% CI = 1.1–5.1, *p* = 0.03) show a positive correlation.

The area under the concentration–time curve was also influenced by some SNPs in our population. The presence of a variant allele *ABCB1* rs9282564 (OR = 4.2, 95% CI = 0.9–18.2, *p* = 0.04) was associated with higher AUC_0-48_ values. In the case of the variant allele *ABCC3* rs4793665 (OR = 0.2, 95% CI = 0.1–0.9, *p* = 0.03), as well as the homozygous variant genotype allele *ABCC2* rs3740066 (OR = 0.1, 95% CI = 0.01–0.8, *p* = 0.01), AUC_0-48_ was lower.

The peak MTX concentrations proved to be higher in the presence of the polymorphism *ABCB1* rs9282564 (OR = 8.8, 95% CI = 1.02–75.6, *p* = 0.02).

For *ABCB1* rs9282564, the AUC_0-48_ of the concentration–time curve and the peak concentration were higher in patients with the variant allele, compared with patients with the homozygous TT genotype.

A higher 48-h MTX concentration value showed a correlation with the presence of variant alleles of *NR1I2*, rs3732361 (OR = 2.6, 95% CI = 1.2–5.9, *p* = 0.01), and rs6785049 (OR = 14.0, 95% CI = 1.8–106.5, *p* = 0.006) SNPs.

### Hepatotoxicity and myelotoxicity

The risk of hepatic toxicity was lower in the presence of variant alleles of *NR1I2* rs3732361 (OR = 0.1, 95% CI = 0.01–0.7, *p* = 0.014), rs3814058 (OR = 0.3, 95% CI = 0.1–0.7, *p* = 0.007), and rs6785049 (OR = 0.1, 95% CI = 0.01–0.7, *p* = 0.02) SNPs.

The risk of bone marrow toxicity was higher due to a mutation in *ABCC2* rs2273697 (OR = 3.3, 95% CI = 1.2–9.4, *p* = 0.02) and lower due to variant alleles of *ABCC2* rs3740066 (OR = 0.4, 95% CI = 0.2–0.9, *p* = 0.02), *NR1I2* rs3732361 (OR = 0.1, 95% CI = 0.01–0.7, *p* = 0.013), rs3814058 (OR = 0.3, 95% CI = 0.1–0.7, *p* = 0.007), and rs6785049 (OR = 0.09, 95% CI = 0.01–0.7, *p* = 0.01) SNPs.

### Bayesian network-based Bayesian univariate and multilevel analysis of relevance (BN-BMLA)

**Figure 2:**
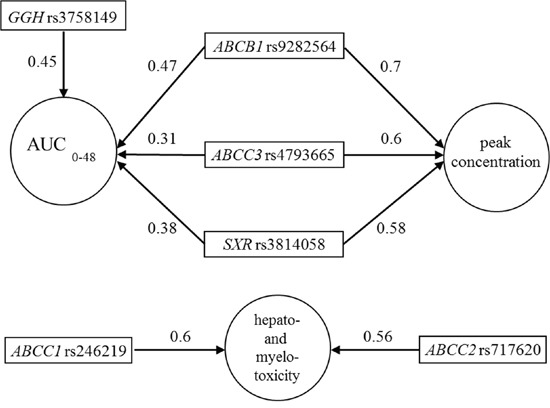
Subgraphs of the strongly relevant variables in pharmacokinetics and hepato-and myelotoxicity according to the univariate Bayesian network-based Bayesian multilevel analysis of relevance (BN-BMLA) The direction of the arrows determines the variable that may produce an effect on the other. Number on the arrow: posterior probability indicating the level of probability that the models may confirm a strong relationship between two variables.

Using the univariate BMLA method, we analyzed whether the above-mentioned 29 SNPs had any effect on the pharmacokinetic factors (AUC_0-48_, peak MTX level, T_α1/2_), as well as the hepatic and bone marrow toxicity. This method gives posterior probability values, indicating the level of probability that the models confirm a strong relationship between the SNP and the analyzed variable, taking into consideration relevant information. The posterior probability is given in brackets after the SNP. The AUC_0-48_ is likely to be influenced by *ABCB1* rs9282564 (0.47), *ABCC3* rs4793665 (0.31), *GGH* rs3758149 (0.45), and *NR1I2* rs3814058 (0.38) (Figure 2). The peak concentration is likely to be influenced by *ABCB1* rs9282564 (0.7), *ABCC3* rs4793665 (0.6), and *NR1I2* rs3814058 (0.58). Hepatic toxicity may be influenced by *ABCC1* rs246219 (0.6), while bone marrow toxicity may be influenced by the presence of *ABCC2* rs717620 (0.56) (Figure 2).

**Figure 3:**
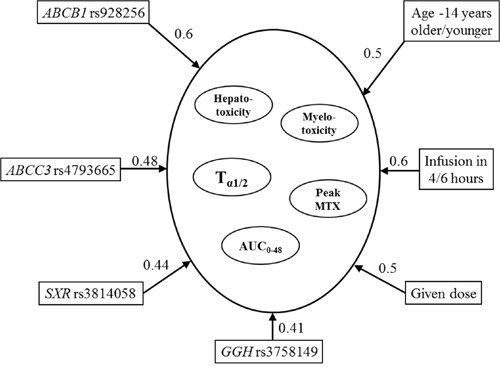
Subgraphs of the strongly relevant variables in pharmacokinetics and hepato- and myelotoxicity according to the multivariate BN-BMLA The direction of the arrows determines the variable that may produce an effect on the other. Number on the arrow: posterior probability indicating the level of probability that the models may confirm the strong relationship between two variables.

In multiple-target variable BMLA analysis, beyond all possible inner interrelations of the cluster of the target variables (pharmacokinetic and toxicity data) and explanatory variables (age, gender, applied dose, duration of infusion, and the 29 SNPs), we also examined which explanatory variables will have direct relevance for the cluster of target variables. A relevant effect for the target variable cluster was detected in the case of *ABCB1* rs928256 (0.6) SNP (Figure 3), which affected the AUC_0-48_ and also the peak concentration according to univariate analysis. Similarly, in case of the *ABCC3* rs4793665 (0.48) and *GGH* rs3758149 (0.41) SNPs, the influence on the area under the curve could also be demonstrated using multivariate analysis. In the multivariable BMLA analysis, *NR1I2* rs3814058 (0.44) may also influence the network system of toxicity and pharmacokinetics; using the univariate BMLA method, it was found to be related to the pharmacokinetic data, and the univariate association method showed a correlation with toxicity occurrence.

## DISCUSSION

There are inter-individual differences in the pharmacokinetics of high-dose MTX treatment, which may lead to toxicities. Due to the complex metabolism and pathway of MTX, there are still several unknown factors that are likely to be revealed through further research. Genetic variants of genes involved in transport and metabolism were analyzed in patients with osteosarcoma to study the pharmacogenetics, pharmacokinetics, and toxicities of MTX.

Certain factors that distort MTX metabolism are already known and proven, such as hydration, pH level of urine, leucovirin dosage, anatomic and physiological parameters, and differences in the protocol, along with many other factors. Beyond these, individual differences in pharmacokinetics and toxicity are mainly influenced by genetic factors, but the significance of these factors regarding their influence in MTX metabolism has yet to be determined.

The most frequently studied polymorphism of the *GGH* gene is SNP -401C>T, located in the promoter region regulating gene expression. This SNP influenced the area below the 48-h curve in our population, identified using univariate and multivariate BN-BMLA analysis. The polymorphism in the *GGH* -401C>T promoter and the MTX polyglutamate levels in red blood cells were studied in patients with rheumatoid arthritis, who were treated weekly with low-dose MTX. The probability to have a MTXPG3-5 level below the group median was 4.8 times higher in patients with the *GGH*-401TT genotype, compared with patients with the C allele [22]. The enzyme GGH is important in both folate and antifolate metabolism; therefore, it is difficult to clearly define its direct role in antifolate drug resistance [23]. The influence on folic acid metabolism may compensate its effect on MTX [24]. The overexpression of the enzyme reduces the effect of antifolates, as the cells are unable to accumulate the polyglutamated form [22]. However, due to the increased GGH activity, folic acid tends to be released faster, which may cause an increased sensitivity to antifolates [10]. Cole et al. found that the overexpression of the enzyme GGH did not provide resistance to short-term MTX exposure in cancer cells. As well as affecting MTX metabolism and accumulation, the enzyme influences folic acid metabolism, which may compensate its effect on MTX [24]. The enzyme GGH is evidently important in the folate and antifolate metabolism in cells; however it is difficult to clearly define its direct role in antifolate drug resistance. Nevertheless, the measurements of the levels of GGH may provide valuable information in determining the antifolate sensitivity of tumors [23].

ABC-transporters are important in drug adsorption, distribution, and elimination. Genetic variants of six ABC-transporter genes were studied to assess whether they have an effect on the pharmacokinetics and toxicity of MTX. The AUC_0-48_ of the concentration–time curve and the peak concentration proved to be higher in patients with the *ABCB1* rs9282564 variant allele, according to both the frequentist-based univariate and univariate BN-BMLA analysis. In our analysis with multiple target variables, the studied *ABCB1* SNP was also shown to have a relevant effect on the target variables combining the kinetics and toxicity. The rs9282564 (Asn21Asp) SNP of ABCB1 is a missense polymorphism and creates a T>C exchange at position 61 within exon 2 of the gene. The role of this polymorphism has been studied in connection with several drugs, but no reduced drug elimination has been observed in rs9282564CC homozygous HeLa cell lines [25]. Also, Kimchi-Sarfaty et al. not could detect a reduced gene expression in the presence of this variant in HeLa cell line [25]. In the literature, there are no studies that examine the correlation between this polymorphism and the pharmacogenetics of MTX. Sainz et al. studied the polymorphism in connection with the formation of colorectal tumors and detected a significant association between its presence and the risk of colorectal tumors in men [26].

The rs4793665 polymorphism of the *ABCC3* gene was relevant in both univariate and the multivariate analyses. It is located in the promoter region of the gene and causes a C>T exchange at base-pair-211. *ABCC3* was studied by sequencing 103 healthy Caucasian individuals, searching for genetic variants which may be related to the mRNA and protein levels measured in the liver. The mRNA level and the protein level of the ABCC3 gene did not correlate with each other. However, in individuals with the -211TT genotype, the mRNA level of ABCC3 was significantly lower compared with people with the CC and CT alleles. It has also been shown that the presence of the polymorphism influences the binding of nuclear factors [27]. In patients with juvenile idiopathic arthritis treated with MTX, *ABCC3* rs4793665 had significant effects on the therapeutic response. It could be reached more frequently in the presence of the variant allele (T), compared with patients with other genotypes [28].

NR1I2, also known as SXR, induces the expression of other enzymes or transporters, such as CYP3A4, CYP3A5, and ABCB1 [29]. The *NR1I2* variant rs3814058 was shown to be important in all three of the analyses in our population, specifically: (1) it influenced the risk of bone marrow and liver toxicity using univariate frequentist-based analysis and showed a strong relevance with the AUC_0-48_ and with the peak concentration in univariate BN-BMLA analysis and (2) it had an influence on pharmacokinetic toxicity in multivariate BN-BMLA analysis. *NR1I2* rs3814058, located in the 3′- untranslated region of the gene, causes a T>C base-pair replacement at position 42961 UTR, and is poorly documented in the literature. In a Scandinavian study, the prognostic role of the *NR1I2* gene variants were examined in primary sclerosing cholangitis; none of the *NR1I2* variants were a predisposing factor to the disease, but patients with the rs3814058 homozygous variant genotype had a lower median survival [30]. The P-glycoprotein (ABCB1) level was 1.45 times lower in bowel biopsy samples of patients with *NR1I2* rs3814058 heterozygous genotype than in samples of patients with the homozygous wild-type genotype [21].

Our results show that several genetic variants may have an impact on MTX kinetics and toxicity in osteosarcoma patients. The metabolic pathway of the metabolism of MTX is very complex; the genetic variants may influence several molecules. Identifying a single genetic difference is not sufficient for a proper prognostic factor of the kinetics and toxicity of MTX. To properly understand the role of genetic variation, a large prospective study examining the effects of several SNPs is recommended. Evaluating the significance of pharmacokinetic factors, the epigenetic, posttranslational, and biochemical differences of MTX metabolism should also be taken into consideration.

## MATERIALS AND METHODS

### Patients

**Figure 4:**
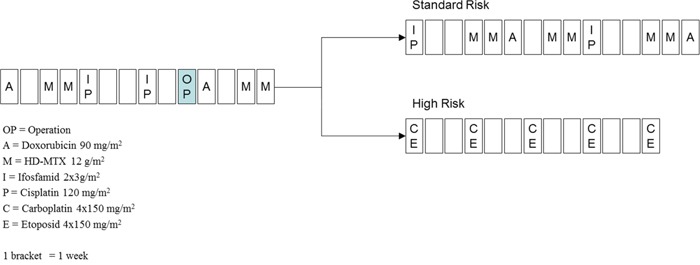
Treatment of osteosarcoma according to the COSS96 protocol A: doxorubicin 90 mg/m^2^; C: carboplatin 4 × 150 mg/m^2^; E: etoposid 4 × 150 mg/m^2^; I: ifosfamide 2 × 3 g/m^2^; M: high-dose methotrexate 12 g/m^2^; OP: date of operation; P: cisplatin 120 mg/m^2^; 1 bracket = 1 week.

Our patient population consisted of 59 children diagnosed with osteosarcoma between 1988 and 2006 at the 2^nd^ Department of Pediatrics of Semmelweis University. All study subjects were Hungarian. Informed consent was requested from the study subjects, or from their parents. The study was conducted according to the principles expressed in the Declaration of Helsinki and was approved by the Hungarian Scientific and Research Ethics Committee of the Medical Research Council (ETT TUKEB; Case No.:8-374/2009-1018EKU 914/PI/08.). Treatment of the patients was based on the COSS-86 and COSS-96 protocols (Figure 4). MTX was used at a dose of 12 g/m^2^ body surface area, 4–12 times during a protocol. The following clinical parameters were collected retrospectively from patients’ charts: age at diagnosis; gender; classification of patients in risk-groups; serum MTX levels measured at 6, 24, 36, and 48 h subsequent to the application of MTX; lowest serum total protein; white blood cell (WBC) and neutrophil granulocyte numbers; highest values of alanine aminotransferase (ALAT); aspartate aminotransferase (ASAT); and bilirubin and creatinine measured during the first two weeks following the MTX treatment. The adverse drug reactions were graded according to the Common Terminology Criteria for Adverse Events v3.0 (CTCAE).

http://www.eortc.be/services/doc/ctc/ctcaev3.pdf

The following target variables were used to describe the pharmacokinetics of MTX: the area under the concentration–time curve (AUC_0-48_), the peak MTX, and the 48-h MTX levels. Two half-lives of MTX excretion were calculated from the MTX serum levels, according to the two-compartment excretion model. The main characteristics of the patients and the detailed description of the toxicities and pharmacokinetic parameters are shown in Table 3.

**Table 3:** Main characteristics of the patients and description of the toxicities and pharmacokinetic parameters

Variable		
Patients	(number)	59
Gender	Male (number)	31
	Female (number)	28
Age at diagnosis	Mean (± SD) (years)	13.6 (± 3.2)
	Median (range) (years)	14.3 (5.5–18.5)
Risk groups	Standard risk (number)	48
	High risk (number)	11
Hepatotoxicity	Grade 1-2 (ASAT, ALAT < 200 U/L) (number)	28
	Grade 3-4 (ASAT, ALAT > 200 U/L) (number)	31
Myelotoxicity	Grade 1 (WBC > 3.0 × 10^-9^/L) (number)	28
	Grade 2-4 (WBC < 3.0 × 10^-9^/L) (number)	29
	Myelotoxicity not known (number)	2
Peak MTX	Median (range) (μmol/L)	1110 (736–1589)
48-h MTX	Median (range) (μmol/L)	0.36 (0.19–1.06)
AUC_0-48_	Median (range) (μmol/L)	13680 (8904–20002)
T_α1/2_	Median (range) (hours)	2.52 (1.9–3.5)

ALAT: Alanine aminotransferase

ASAT: aspartate aminotransferase

AUC _0-48_: area under the concentration–time curve 0–48 h

MTX: methotrexate

SD: standard deviation

T_α1/2_: first half-life time of MTX excretion

WBC: white blood cells

### SNP selection, DNA extraction, and genotyping

DNA was isolated from the blood of children with osteosarcoma using Qiagen isolation kits (QIAmp DNA Blood Maxi Kit or QIAmp DNA Blood Midi Kit, Qiagen, Hilden, Germany). SNPs were selected based on the international literature and prioritized on the basis of their estimated functionality, using the following order: non-synonymous SNPs, SNPs in the promoter and 3′-UTR (3′-untranslated region) region, synonymous SNPs, and intronic SNPs. The minor allele frequency was set at >10%. Our goal was to cover every haplotype block in the gene defined by the Haploview 4.1 software (http://www.broad.mit.edu/mpg/haploview/) (Barrett et al., 2005) with 1 or 2 SNPs. Altogether, 29 single-nucleotide polymorphisms of nine genes (*ABCB1*, *ABCC1*, *ABCC2*, *ABCC3*, *ABCC10*, *ABCG2*, *GGH*, *SLC19A1*, and *NR1I2*) were chosen; detailed information on the selected SNPs are shown in Table 1.

SNPs were genotyped using the GenomeLab SNPstream genotyping platform (Beckman Coulter), according to the manufacturer's instructions. A detailed description of this procedure can be found in our previous article [31].

### Statistical methods

Allele frequencies were calculated by allele counting. The Hardy–Weinberg equilibrium was tested using a χ^2^ goodness-of-fit test with an acceptable cut-off value of *p* ≥ 0.05 (https://ihg.gsf.de/cgi-bin/hw/hwa1.pl).

Univariate association analysis was used to determine whether the presence of the variant allele of SNP associates with an increase or decrease in the value of the pharmacokinetic target variable or the frequency of the toxicity. The chi-square (χ^2^) test was applied. The statistical tests were performed by IBM SPSS statistical software (version 15.1, IBM Corp., Armonk, NY, USA).

BN-BMLA was also used to study the effect of genetic background on pharmacokinetics and toxicity. A Bayesian network is a probabilistic graphical model that represents the conditional dependencies of a set of random variables with a directed acyclic graph. It can efficiently describe the joint probability distribution of the variables. A node of the graph represents a variable, and an edge represents a direct dependency between the corresponding variables. The BN-BMLA method calculates the posterior probability of certain structural features of Bayesian networks (e.g., the presence of an edge between two variables) learned from the data. The current implementation of BN-BMLA deals with only discrete variables, therefore continuous pharmacokinetic covariates must be discretized first, to include them in the BN-BMLA analysis. These variables were discretized based on the median of the values [32], [33]. The posterior probability of the strong relevance of each SNP with respect to the analyzed variable was calculated by the method. A relevant relationship was defined when the probability was >0.3. The applied method was developed by our research team; for further details see the section in the book “Probabilistic graphical models for genetics” [34].

## CONCLUSIONS

In our pharmacogenetic analysis, we used three different statistical methods to identify a correlation between the genotype and the clinical data. SNPs *ABCB1* rs928256, *ABCC3* rs4793665, *GGH* rs3758149, and *NR1I2* rs3814058 were shown to have a relevant effect in both univariate and the multivariate analyses. Pharmacokinetic and pharmacogenetic data would allow developing a population pharmacokinetic model, which could forecast the probable pharmacokinetic parameters individually prior to MTX therapy, thus ensuring an individual medication dosage for each patient.
